# Plectin, a novel regulator in migration, invasion and adhesion of ovarian cancer

**DOI:** 10.1186/s13578-025-01349-2

**Published:** 2025-02-06

**Authors:** Lanning Bai, Xueqian Qian, Hui Zhang, Yi Yuan, Xiaodong Cui, Min Cheng, Yangyang Han

**Affiliations:** 1 School of Basic Medicine Sciences, Shandong Second Medical University, Weifang, Shandong 261053 P. R. China; 2 School of Life Science and Technology, Shandong Second Medical University, Weifang, Shandong 261053 P. R. China; 3 Department of Physiology, Shandong Second Medical University, Weifang, Shandong 261053 P. R. China

**Keywords:** Adhesion, Diagnostic biomarker, Ovarian cancer, Plectin, Therapeutic target

## Abstract

**Background:**

Ovarian cancer (OC) is one of the most prevalent gynecologic malignancies and exhibites the highest fatality rate among all gynecologic malignancies. The absence of an early diagnostic biomarker and therapeutic target contributes to an overall 5-year survival rate ranging from 30 to 50%. Plectin (PLEC), a 500 kDa scaffolding protein, has gained prominence in recent years due to its pivotal role in various cellular biological functions such as cell morphology, migration and adhesion, while the accurate role of PLEC in OC remains elusive.

**Results:**

In this study, our findings demonstrate that PLEC exerts a positive influence on the progression of OC, encompassing cellular proliferation, migration, invasion, and adhesion both in vitro and in vivo.

**Conclusions:**

The results providing new insights for the diagnosis and treatment in OC.

**Supplementary Information:**

The online version contains supplementary material available at 10.1186/s13578-025-01349-2.

## Introduction

Ovarian cancer (OC) is an aggressive and progressive gynecological tumor [1], epithelial ovarian cancer (EOC), one of the most common types of ovarian cancer, which constitutes 90% of the malignant ovarian cancer, result in 19,880 estimate new cases and 12,810 estimated deaths in 2022 in United States [2, 3]. The progression of OC often gives rise to metastasizes, thereby resulting in an unfavorable prognosis and elevated mortality rate among [4]. Although the utilization of a combination of biomarkers and multiple clinical factors has been demonstrated to enhance diagnostic accuracy [5], the prognosis of OC remains poor, with an overall 5-year survival rate ranging from 30–50% [6]. The prognosis of OC remains unfavorable due to the absence of early diagnostic makers.

Plectin (PLEC), a 500 kDa scaffolding protein, abundantly expressed in a variety of mammalian tissues and cells [7], consist of a globular domain at the amino terminus, a globular domain at the carboxy terminus and an α-helical rod-like central domain of approximately 200 nm in length connected therebetween [8] It interlinks intermediate filaments with microtubules and microfilaments and anchors intermediate filaments to desmosomes or hemidesmosomes to affect cell adhesion, migration, proliferation, and other biological functions [9–12]. It has recently been implicated as a pro-tumorigenic regulator of cancer cell biology functions [13]. However, the majority of previous research on PLEC has predominantly focused on its role in skin and skeletal muscles diseases [14]. Although research on the role of PLEC in cancer is now gradually beginning [15], with limited investigations into its involvement in ovarian cancer.

In this study, we investigated the impact of PLEC expression on the progression of EOC by modulating cellular proliferation, migration, invasion, and adhesion both in vitro and *in vivo.* Furthermore, we conducted an extensive analysis to elucidate the underlying mechanisms involved. Our findings suggest that PLEC has potential as an early diagnostic biomarker and therapeutic target of EOC, providing novel insights into its diagnosis and treatment.

## Materials and methods

### Cell culture

Human epithelial ovarian cancer cell lines SKOV3 and A2780 were obtained from Procell (Wuhan, Hubei, China) and FENGHUISHENGWU (Changsha, Hunan, China). The SKOV3 and A2780 cells were cultured in MCCOY’S 5 A medium (Solarbio, Beijing, China) with 15% fetal bovine serum (FBS, TIANHANG, China) or 1640 medium (Hyclone, Cytiva, America) with 10% FBS receptively, which both containing 1% penicillin-streptomycin (Solarbio, Beijing, China). Both cells were routinely grown in a humidified incubator with 5% CO_2_ at 37℃.

### Lentivirus infection

PLEC-Knock Down (KD) cell lines (SKOV3 and A2780) were generated using lentivirus vectors with PLEC shRNA sequence (5’-GGATCCGGTCTCAGTTCCTGAAGTTTATTCAAGAGATAAACTTCAGGAACTGAGACCTTTTTTGAATTC-3’) according to the manufacturer’s instruction, and NC shRNA lentivirus vectors were used as control. Generally speaking, lentivirus vectors were added at a multiplicity of infection (MOI) of 10 into the cells with polybrene when the cells reached 70% confluency, then the medium was replaced after 24 h of infection and the efficiency of transfection was evaluated by western blotting. The PLEC-KD cells were screened with 0.75 µg/ml Puromycin dihydrochloride.

### siRNA transfection

The protocol followed our previous paper [16].The siRNAs targeting COL17A1 and ITGβ4 were designed and synthesized by Sangon Biotech (Shanghai, China), and the knockdown efficiency was assessed using Western blot. A2780 and SKOV3 cells that were transfected with negative siRNA were used as controls, and non-siRNA were used as blank, siRNA1151, siRNA454 inA2780 and siRNA492, siRNA114 in SKOV3 (renamed as KD ITGβ4 and KDCOL17A1) were chosen for further experiment.

### Western blotting

Total protein preparation for Western Blot was performed as described previously [16]. Generally, proteins were extracted using Column Tissue & Cell Protein Extraction Kit (Epizyme, Shanghai, China), boiled in 1×SDS PAGE loading buffer (RB005-001, Rui Biotech, China), and then separated on 10% SDS-PAGE gels. Immunoblotting was performed with primary antibodies against plectin (ab32528, Abcam, UK) and β-actin (AB0501, Abways, Shanghai), then incubated with AffiniPure Goat Anti-Rabbit IgG H&L/HRP (bs-40295G-HRP, Bioss, Beijing) and visualized using an Omni-ECL™Femto Light Chemiluminescence Kit (SQ201, Epizyme, Shanghai).

### Quantitative RT-PCR

Total RNA kit (BSC52M1, BioFlux, China) was used for RNA extraction, and the ReverTra Ace^®^ qPCR RT Kit (FSQ-101, TOYOBO, Shanghai) was used for cDNA synthesize. PCR was carried out in a 20 µl reaction containing 1×SYBR Green PCR Master Mix (AQ601, TransGen, China) with conditions of 95℃ for 3 min, 95℃ for 30 s, 60℃ for 30s and 72℃ for 30s for 40 cycles. All the procedures were carried out according to each manufacturer’s instructions.

Primer sequences for PLEC are:

5’-CCGCCTCTTCAATGCCATCATCC-3’(plectin-F),

5’-TCCAGGTTCTCCAGGTTGGTCTG-3’ (plectin-R).

Primer sequences for GAPDH are:

5’-CAGGAGGCATTGCTGATGAT-3’ (GAPDH-F),

5’-GAAGGCTGGGGCTCATTT-3’ (GAPDH-R).

### Colony formation

Cells were seeded into 6-well plates at 1 × 10^3^/well dispersedly and cultured in medium till the cells of majority individual clones have greater than 50 under microscope. Fixed the cells in 4% paraformaldehyde at 20 min, stained by 0.1% crystal violet, and record with camera. All the experiments were repeated 3 times.

### CCK-8 assay

Cells were seeded into 96-well plates at 5 × 10^3^/well and cultured in complete medium for 24/48 h, then 10 µl CCK-8 solution (Proteintech, Wuhan, China) was add into each well according to the manufacturer’s instruction. The OD value of 450 nm was determined 4 h later.

### Wound healing assay

Cells were seeded into 6-well plates and cultured in complete medium till the cell density reaches about 90%, then scratched vertically along the edge of ruler using 20 µl tips, wash 2 times with PBS, and add the medium with 2% FBS. Results were observed and recorded at 0, 24 and 48 h after scratching using light microscope (CKX53, Olympus, Japan). All the experiments were repeated 3 times.

### Cell migration and invasion assays

Cells were seeded into 24-well transwell upper chambers (3422, Corning, USA) at 5 × 10^3^/chamber with serum-free medium, and the lower chambers were filled with 600 µl of complete medium. 100 µg Matrigel (354234, Corning, USA) was precoated at the end of the chamber in the invasion assay. Then fixed the cells in 4% paraformaldehyde at 24 h, stained by 0.1% crystal violet, and recorded using light microscope (CKX53, Olympus, Japan). All the experiments were repeated 3 times.

### Dissociation assay

The protocol was described clearly in previous paper [17]. Briefly, Cells were seeded into 6-well plates at proper density, cultured in complete medium for 24 h. Cells were then separated from the plates with Trypsin-EDTA Solution (Solarbio, Beijing, China). Transfer the dispase solution into a new 6-well plate, next, the cell was mechanically stressed by pipetting up and down 7 times carefully.

### Adhesion assay

The adhesion capacity of the cells was measured by cell adhesion detection kit (BestBio Company, Shanghai, China) according to the manufacture’s instruction. Briefly, the coating liquid precoated at the 96-well plates overnight at 4℃, seeded cells into 96-well plates at 5 × 10^3^/well, 37 °C incubator for 30 min. Then added 20 µl staining solution B to cells, and value the OD at 450 nm wavelength at 2 h later.

### Confocal microscopy assay

Cells were seeded into confocal dishes at 5 × 10^3^/well, cultured in complete medium, then fixed the cells in 4% paraformaldehyde at 24 h. Target proteins were visualized using primary antibodies against plectin (ab32528, Abcam, UK), collagen XVII (COL17A1, ab184996, Abcam, UK), integrin beta4 (ITGβ4, ab182120, Abcam, UK), and then secondary antibodies including Cy3-labeled Goat Anti-Rabbit IgG (H + L) (A0516, Beyotime, China), FITC-labeled Goat Anti-Rabbit IgG (H + L) (A0562, Beyotime, China). Nuclei were stained by 4′,6-diamidino-2-phenylindole (DAPI). The images were recorded under a confocal laser scanning microscope (TCS SP8, Leica, Germany).

### Immunohistochemical analysis

Epithelial ovarian cancer tissue microarrays (Ova-809, Alenabio, China) were purchased for PLEC detecting. Paraffin sections were dewaxed and hydrated. Antigen were retrieved by citrate buffer and blocked H_2_O_2_ (PV-9003, Goat two-step test kit, ZSGB-BIO) at 37 °C for 20 min, and blocked at room temperature with 1% BSA for 1 h. Slides were incubated with diluted primary antibodies (1:500) including plectin (ab32528, Abcam, UK) and COL17A1 (ab184996, Abcam, UK) and ITGβ4 (ab182120, Abcam, UK) at 4 °C overnight followed by secondary antibody of the Goat two-step test kit for 20 min at room temperature. The slides were stained by diaminobenzidine (DAB) of the Goat two-step test kit and staining in hematoxylin solution for 2 min, mounted by 1% hydrochloric acid alcohol differentiation solution. Pictures were taken by 20× magnification light microscope (U-HGLGPS, Olympus, Japan). The H-Score of each spot on each chip was quantified using the Densito quantitative module of Quant Center 2.1 analysis software, and the H-Score score represents the strength of positive expression. And the details of ovarian tissues are shown in Table 1.

**Table 1:** Details of ovarian tissues

Pos.	Age	Pathology diagnosis	TNM	Type	H-Score
A1	42	Mucinous adenocarcinoma	T1bN0M0	malignant	104.53
A2	30	Serous papillary adenocarcinoma	T1N0M0	malignant	143.51
A3	40	Serous papillary adenocarcinoma	T1N0M0	malignant	114.55
A4	43	Serous papillary adenocarcinoma	T1N0M0	malignant	114.47
A5	60	Serous papillary adenocarcinoma	T2N0M0	malignant	118.61
A6	57	Serous papillary adenocarcinoma	T1cN0M0	malignant	42.19
A7	39	Serous papillary adenocarcinoma	T1aN0M0	malignant	126.92
A8	53	Serous adenocarcinoma	T1aN0M0	malignant	100.44
A9	39	Serous adenocarcinoma	T1aN0M0	malignant	83.83
A10	48	Endometrioid adenocarcinoma	T1N0M0	malignant	124.96
B1	45	Endometrioid adenocarcinoma	T2N0M0	malignant	110.34
B2	70	Transitional cell carcinoma	T1N0M0	malignant	132.54
B4	68	Clear cell carcinoma	T1N0M0	malignant	76.52
B5	37	Clear cell carcinoma	T1aN0M0	malignant	75.05
B6	48	Mucinous adenocarcinoma	T3aN1M1	malignant	42.19
B8	38	Mucinous papillary adenocarcinoma	T2bNxM0	malignant	63.34
B9	35	Mucinous papillary adenocarcinoma	T3aN1M0	malignant	64.6
B10	34	Mucinous adenocarcinoma	T2cN1M0	malignant	96.41
C1	50	Mucinous adenocarcinoma	T3cN1M0	malignant	85.33
C4	38	Serous papillary adenocarcinoma	T2bN0M0	malignant	119.7
C5	43	Serous papillary adenocarcinoma	T3cN1M0	malignant	76.82
C6	43	Serous papillary adenocarcinoma	T2bN1M0	malignant	67.34
C7	49	Serous papillary adenocarcinoma	T2cNxM0	malignant	64.55
C8	59	Serous adenocarcinoma (fibrous tissue and blood vessel)	T2N0M0	malignant	67.37
C9	70	Serous adenocarcinoma	T2bN0M0	malignant	58.44
C10	64	Serous papillary adenocarcinoma	T2N1M0	malignant	78.3
D1	51	Serous papillary adenocarcinoma	T2N1M0	malignant	70.41
D2	47	Serous papillary adenocarcinoma	T3cN1M0	malignant	100.4
D3	59	Serous papillary adenocarcinoma	T3aN0M0	malignant	35.48
D4	49	Serous adenocarcinoma	T3aN1M0	malignant	56.54
D5	76	Serous papillary adenocarcinoma	T2cN0M0	malignant	26.19
D6	37	Serous papillary adenocarcinoma	T3bN1M0	malignant	31.37
D7	52	Serous papillary adenocarcinoma	T3aN0M0	malignant	34.54
D8	47	Serous papillary adenocarcinoma	T3bN1M0	malignant	99.73
D9	48	Serous papillary adenocarcinoma	T2bNxM0	malignant	111.81
D10	50	Serous papillary adenocarcinoma	T3aN0M0	malignant	66.67
E1	51	Serous adenocarcinoma	T3N1M1	malignant	125.85
E2	43	Serous papillary adenocarcinoma	T3N1M0	malignant	44.06
E3	53	Serous papillary adenocarcinoma	T3N0M0	malignant	67.07
E4	39	Serous adenocarcinoma	T3cN1M0	malignant	32.56
E5	53	Serous papillary adenocarcinoma	T3bN1M0	malignant	29.3
E6	48	Serous papillary adenocarcinoma	T2N1M0	malignant	66.38
E7	47	Serous papillary adenocarcinoma with necrosis	T3bN0M0	malignant	35.26
E9	43	Serous papillary adenocarcinoma	T3cN1M0	malignant	104.67
E10	63	Serous adenocarcinoma	T2N1M0	malignant	45.46
F1	56	Serous adenocarcinoma	T2N1M0	malignant	52.95
F2	48	Serous adenocarcinoma	T3aN1M0	malignant	73.37
F3	63	Serous papillary adenocarcinoma	T3cN1M0	malignant	84.35
F4	60	Serous papillary adenocarcinoma	T2bN0M0	malignant	99.03
F5	49	Serous papillary adenocarcinoma	T3bN1M0	malignant	75.59
F6	63	Serous adenocarcinoma	T2N1M1	malignant	61.17
F7	42	Serous adenocarcinoma	T2bN0M0	malignant	99.98
F8	49	Serous adenocarcinoma	T3cN1M0	malignant	83.64
F9	64	Serous papillary adenocarcinoma	T3cN1M0	malignant	47.01
F10	66	Serous adenocarcinoma	T2N1M0	malignant	111.3
G2	40	Serous adenocarcinoma	T2N1M0	malignant	62.89
G3	46	Serous adenocarcinoma	T2N1M0	malignant	79.87
G4	54	Serous adenocarcinoma	T2N1M0	malignant	50.28
G5	54	Serous papillary adenocarcinoma	T3cN0M0	malignant	84.05
G6	51	Serous adenocarcinoma	T2bNxM0	malignant	94.33
G7	54	Serous adenocarcinoma	T3cN1M1	malignant	90.05
G8	53	Serous adenocarcinoma	T3N1M0	malignant	120.64
G9	40	Endometrioid adenocarcinoma	T2N1M0	malignant	118.42
G10	52	Transitional cell carcinoma	T2N1M0	malignant	90.11
H1	40	Normal ovarial tissue	-	malignant	36.78
H2	19	Normal ovarial tissue	-	normal	47.64
H3	18	Normal ovarial tissue	-	normal	19.6
H4	40	Normal ovarial tissue	-	normal	118.14
H5	21	Normal ovarial tissue	-	normal	111.52
H6	21	Normal ovarial tissue	-	normal	112.06
H7	18	Normal ovarial tissue	-	normal	60.98
H8	15	Normal ovarial tissue	-	normal	61.94
H9	20	Normal ovarial tissue	-	normal	54.58
H10	14	Normal ovarial tissue	-	normal	62.28

### In vivo assays

Female BALB/c nude mice (4-week-old) were purchased (Charles River) and randomized into treatment and control groups (*n* = 5 mice/group) for further in vivo assays. The A2780 cells were chosen to be used in the in vivo assays.

For the analysis of subcutaneous tumor models, a mixture of A2780 cells (1 × 10^8^/ml) and matrigel matrix (354248, Corning, America) was subcutaneously injected into the BALB/c nude mice (100 µl/mouse). Tumor volume was measured every five days with vernier calipers, excised the tumors from the mice once the tumor volume reached tissues approximately 1000–2000 mm^3^. Subsequently, the excised tissues were cut into small pieces measuring approximately 2–3 mm^3^ and implanted in the flank of female mice aged 4-week-old. Size of tumor tissues were measured every 3 days using a caliper.

For the analysis of tumor in situ, 15 µl cells, which carrying a dual GFP/luciferase expression system (KDPLEC) and shEV (Control), were injected into the left ovary of the 4-week-old female BALB/c nude mice at 1 × 10^8^/ml (serum-free medium). The location and numbers of these transduced cells were visualized using IVIS (PE IVIS Spectrum, PerkinElmer) at 5 weeks after the injection.

The resected tumors were fixed in paraffin-embedded blocks for subsequent IHC analysis.

### Statistical analysis

Data were statistically analyzed using GraphPad Prism 9 (GraphPad Software), two-tailed unpaired Student’s t-test. Pictures was analyzed using ImageJ. At least 3 times repeat each experiment, and values represent the mean ± standard deviation of the mean. P values less than 0.05 is considered as a standard of significant difference.

## Results

### The expression of PLEC is highly related with epithelial ovarian cancer

**Fig. 1:**
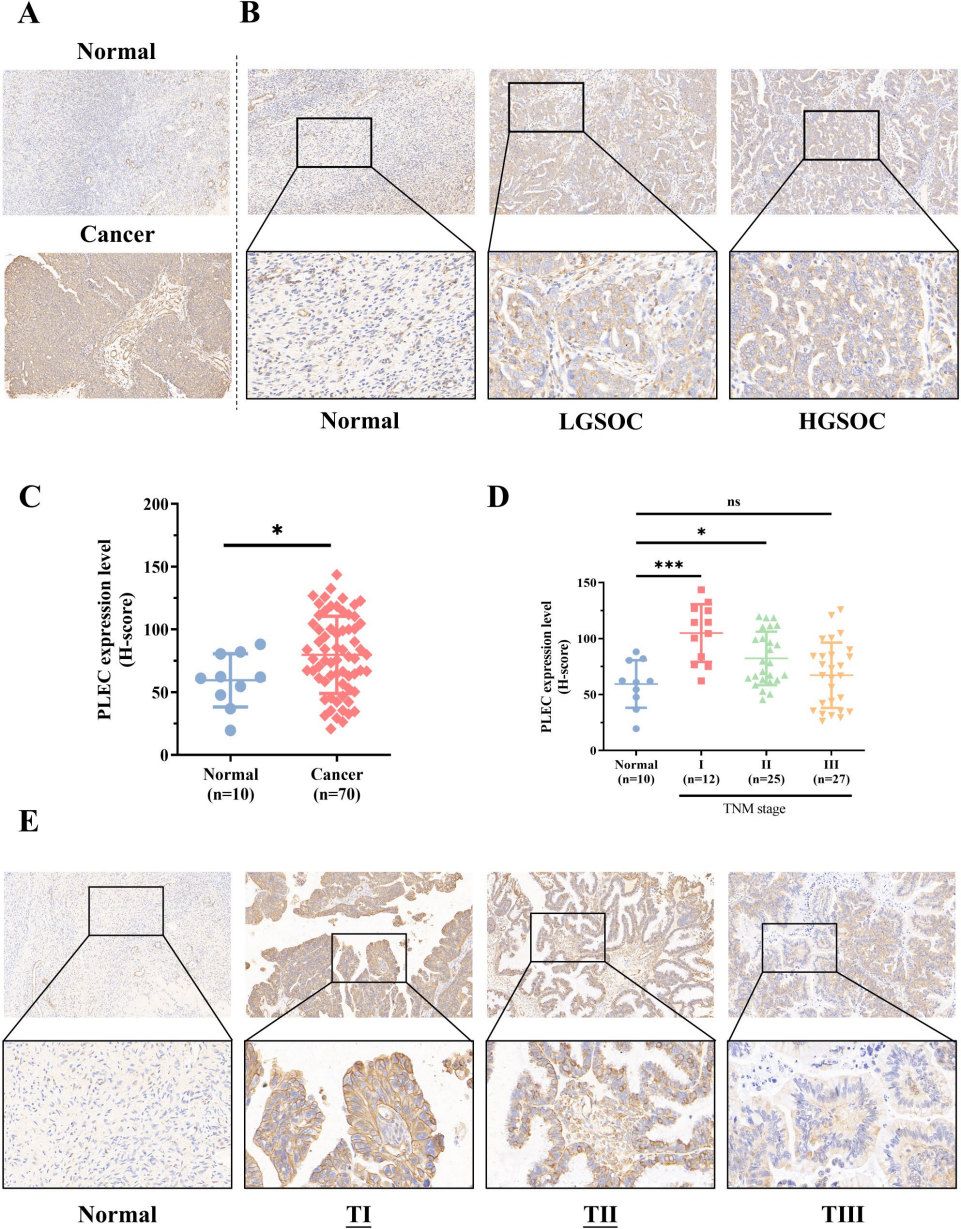
The expression of PLEC is significantly upregulated in EOC. **A** Expression of PLEC in normal and EOC tissues. **B** Expression of PLEC in normal tissues and different types of EOC (LGSOC and HGSOC) tissues. **C** The result of statistical analysis of H-Score expressed by PLEC in A. **D** The result of statistical analysis of the H-Score of PLEC expression in E. **E** The expression of PLEC in normal tissues and EOC tissues of different stages (I, II and III). P-value: *<0.05; ***<0.001; ns: no significance, determined by two-tailed Student’s t-test

Our previous paper has indicated that the mRNA expression of PLEC was upregulated in ovarian cancers compared with that in normal ovarian tissues based on bioinformatics analysis [16]. Further in this paper, our results demonstrated that the protein expression level of PLEC in pathological tissues is higher than that in normal tissue according to the immunohistochemical results (Fig. 1C), and the typical images were shown as in Fig. 1A and B. But as the TNM stages increased, the expression of PLEC did not show a consistent rise according to the H-Score results (Fig. 1D, E). Taken together, our results in Fig. 1 suggested that the protein expression of PLEC was actually higher in epithelial ovarian cancer tissues at the overall level.

### PLEC promoted ovarian cancer cell proliferation in vitro and in vivo

**Fig. 2:**
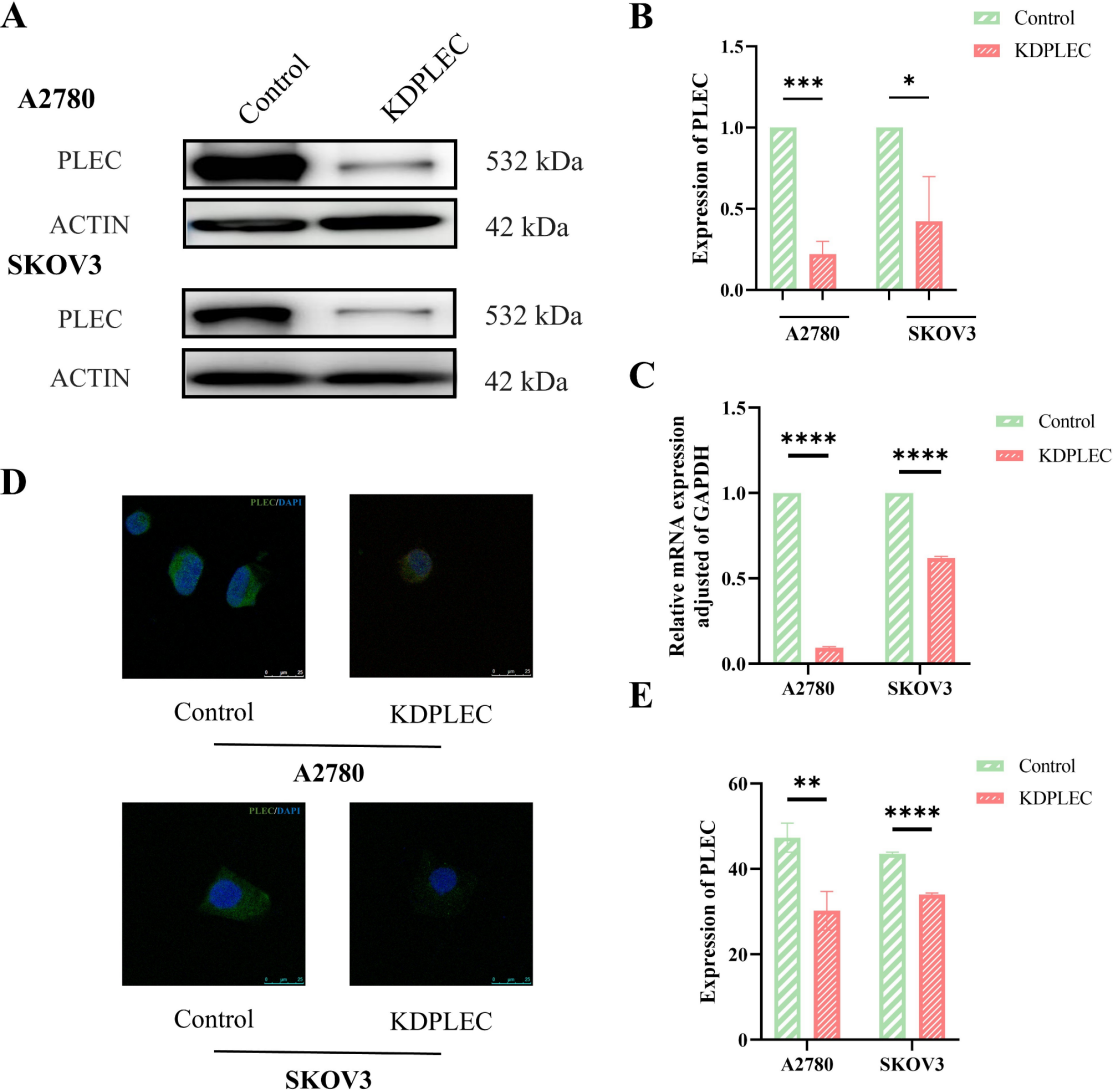
Constructed A2780 and SKOV3 PLEC knock-down and control EOC cell lines. **A** PLEC was knock-down in A2780 and SKOV3. Expression of PLEC was determined by western blotting analysis. **B** The result of statistical analysis of the grayscale value expressed by PLEC in A, quantified by imageJ, error bars represent SD. **C** The result of statistical analysis of expression of PLEC determined by qRT-PCR, error bars represent SD. **D** Confocal assay: A2780 and SKOV3 PLEC knock-down and control EOC cells were seeded into confocal dishes at 5 × 10^3^/well, fixed after 24 h. Target proteins were visualized using antibodies, nuclei were stained by DAPI (green: PLEC, blue: DAPI). **E** The result of statistical analysis of the fluorescence intensity value expressed by PLEC in D, quantified by imageJ, error bars represent SD. P-value: *<0.05; **<0.01; ***<0.001, **** <0.0001, determined by two-tailed Student’s t-test

To demonstrated the accurate role of PLEC in epithelial ovarian cancer, we constructed two PLEC knock-down and Control epithelial ovarian cancer cell lines (A2780, SKOV3) for further research. The results of Western Blot confirmed that PLEC was successfully knock down (Fig. 2A, B), similar with the result of qRT-PCR (Fig. 2C). While confocal assay also revealed pronounced disparities in PLEC fluorescence intensity (Fig. 2D, E).

**Fig. 3:**
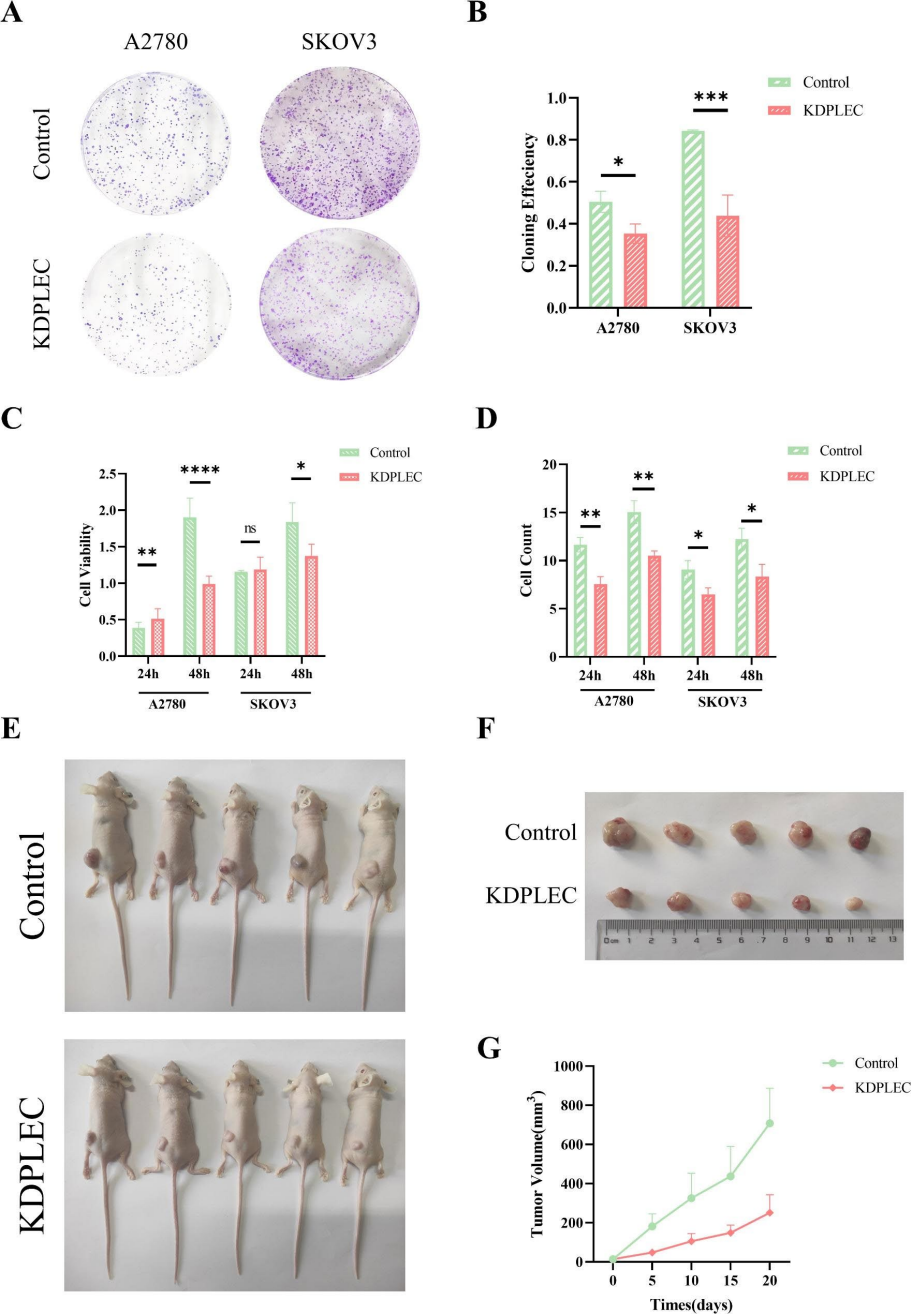
PLEC promoted ovarian cancer cell proliferation both in vitro and *in vivo.***A** Colony formation assays of A2780 and SKOV3 PLEC knock-down and control EOC cells. Cells were fixed 2 weeks later, stained with crystal violet. **B** The result of statistical analysis of the area covered by colonies across average expressed by PLEC in A, quantified by imageJ, error bars represent SD. **C** Cells were seeded into 96-well plates at 5 × 10^3^/well, The OD value of 450 nm was determined at 24/48 h. Error bars represent SD. **D** Cells were seeded into 96-well plates at 4 × 10^3^/well, count the cell number at 24/48 h. **E** Injected the mixture of A2780 PLEC knock-down and control cells (1 × 10^8^/ml) and matrigel matrix subcutaneously into the BALB/c nude mice (*n* = 5) till the tumor volume reached tissues approximately 1000–2000 mm^3^. **F** Excised the tumors from the mice. **G** Tumor volume was measured every five days with vernier calipers, error bars represent SD. P-value: *<0.05; **<0.01; ***<0.001; ns: no significance, determined by two-tailed Student’s t-test

Using the KDPLEC and Control cell lines, we then verified that PLEC played a positive role in the proliferation of EOC cells in vitro. As the results shown in Fig. 3A-D, both two KDPLEC cell lines exhibited significantly decreased colony formation (Fig. 3A, B), cell viability (Fig. 3C) and cell count (Fig. 3D). Similar result was further confirmed in vivo using subcutaneous tumor model, the tumor volume of KDPLEC group is significantly smaller than Control both in vivo (Fig. 3E) and ex vivo (Fig. 3F-G).

### PLEC promoted ovarian cancer cell invasion and metastasis in vitro and in vivo

**Fig. 4:**
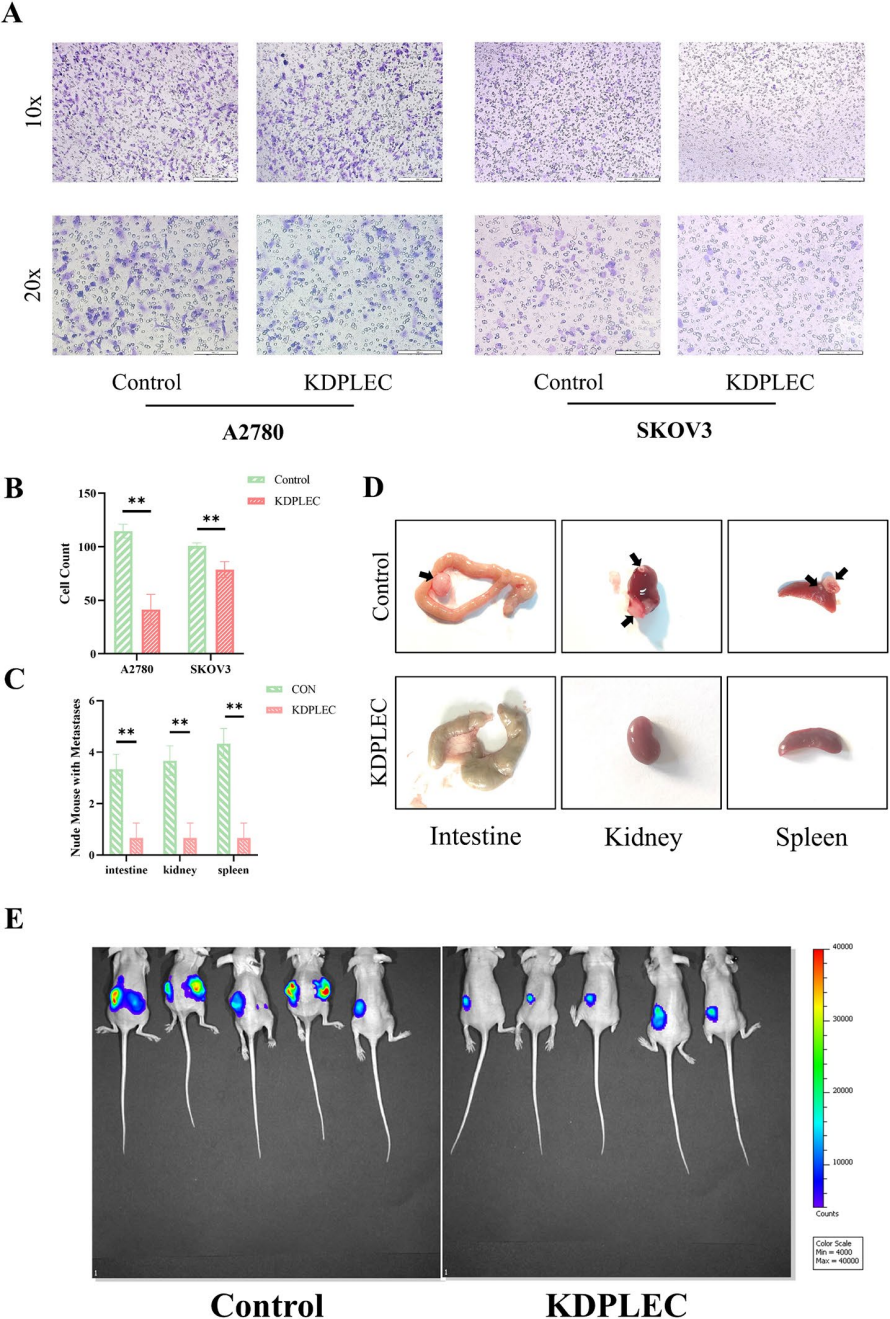
PLEC promoted ovarian cancer cell invasion in vitro and in vivo. **A** Cells were seeded into 24-well transwell upper chambers at 5 × 10^3^/chamber with serum-free medium, and the lower chambers with complete medium. Matrigel was precoated at the end of the chamber, fixed the cells at 24 h, stained by crystal violet. **B** Cell count quantified by imageJ, error bars represent. **C** The statistical analysis of the number of tumor metastasis. **D** Representative intestine, kidney, and spleen tissues of KDPLEC and control. Arrowheads, metastatic tumors. **E** 15 µl KDPLEC and control cells were injected into the left ovary of the BALB/c nude mice at 1 × 10^8^/ml, visualized using the IVIS in vivo imaging system after 5 weeks. Error bars represent SD. P-value: **<0.01, determined by two-tailed Student’s t-test

The invasion capacity of two PLEC knockdown cells was observed to decrease when 100 µg Matrigel was precoated at the end of the chamber (Fig. 4A, B), suggesting that PLEC plays a crucial role in promoting cell invasion capacity in vitro. Meanwhile, the IVIS imaging results demonstrated a significant reduction in the number of metastases in KDPLEC group compared to the Control group in vivo (Fig. 4E), and using visual inspection, a widely accepted method when animals were dissected [18–20], we found that the number of metastatic nodules was significantly lower in intestine, kidney, and spleen of the KDPLEC group than Control group (Fig. 4C), and the typical images were shown as Fig. 4D, which was consistent with our previous findings on cell invasion capacity in vitro.

### PLEC promoted ovarian cancer cell migration and adhesion in vitro

**Fig. 5:**
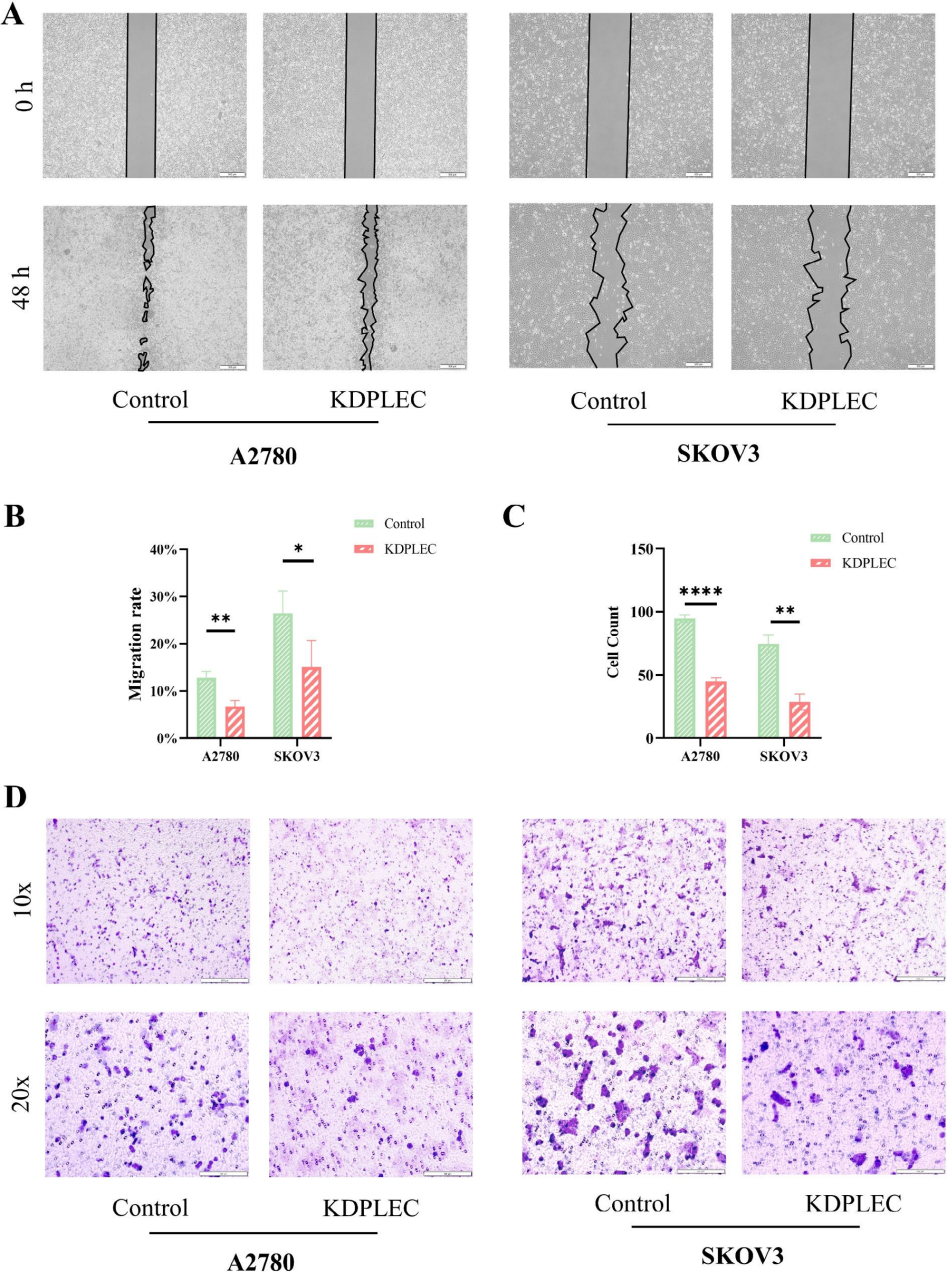
PLEC promoted ovarian cancer cell migration in vitro. **A** Wound Healing Assay of A2780 and SKOV3 PLEC knock-down and control EOC cells, recorded at 0 and 48 h. **B** The area of blank was quantified by imageJ, error bars represent. **C** The result of statistical analysis of the cell counts in D, quantified by imageJ, error bars represent. **D** Cells were seeded into 24-well transwell upper chambers at 5 × 103/chamber with serum-free medium, and the lower chambers with complete medium, fixed the cells at 24 h, stained by crystal violet. P-value: *<0.05; **<0.01; ****<0.0001; ns: no significance, determined by two-tailed Student’s t-test

The impact of PLEC on cell migration was subsequently validated through Wound Healing Assay and Cell Migration Assay. The typical images from the Wound Healing Assay demonstrated a significant increase in the area between two edges of KDPLEC cells compared to the Control cells in both two cell lines, indicating that knocking down PLEC effectively suppressed cell migration at 48 h post-scratching (Fig. 5A), and the results were further confirmed according to the statistical analysis (Fig. 5B and C). Furthermore, the results obtained from the Cell Migration Assay indicated that the migratory cell count of the KDPLEC cells was substantially diminished compared to the Control cells, subsequent to an equivalent duration of cultivation. (Fig. 5D).

**Fig. 6:**
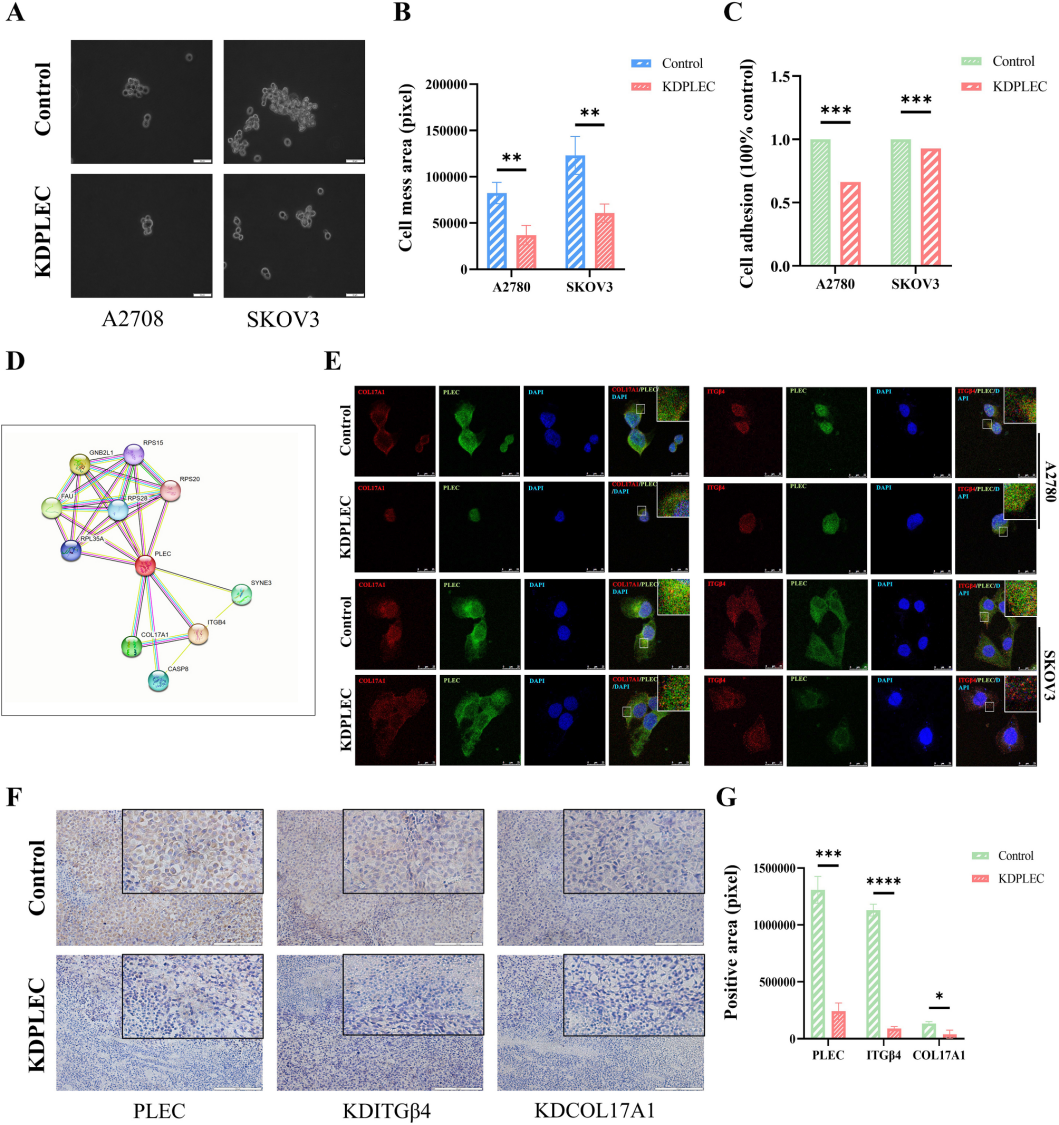
PLEC promoted ovarian cancer cell adhesion in vitro with COL17A1 and ITGβ4. **A** The image fragmented cell monolayers which were detached with dispase and mechanically fragmented by pipetting. **B** The area of those cell fragments quantification by imageJ, error bars represent. **C** pre-coating the 96-well plate with the coating solution from the adhesion detection kit, seeding the cells in the 96-well plate at a density of 5 × 104 cells per well, adding 10 µl of cell staining solution to each well for 2 h after incubation, and subsequently measuring the OD value of the sample wells after 4 h, followed by statistical analysis of the data. **D** COL17A1 and ITGβ4 are highly related with PLEC (https://cn.string-db.org/). **E** Confocal assays (green: PLEC, red: ITGβ4/COL17A1, blue: DAPI). **F** IHC of tumor slides. **G** The result of statistical analysis of the fluorescence intensity value expressed by PLEC, ITGβ4 and COL17A1 in B, quantified by imageJ, error bars represent SD. P-value: *<0.05; **<0.01; ***<0.001; ****<0.0001, determined by two-tailed Student’s t-test

Previous studies have demonstrated a strong association between PLEC and focal adhesion [21, 22], suggesting that PLEC plays a regulatory role in cell adhesion. To validate this hypothesis, we performed a dissociation assay which revealed that knockdown PLEC promoted cell dissociation (Fig. 6A, B). Subsequently, we assessed the adhesion capacity of cells using a cell adhesion detection kit and observed a significant suppression in adhesion capacity following PLEC knockdown (Fig. 6C).

### PLEC modulates cell adhesion through its interaction with COL17A1 and ITGβ4

In an endeavor to delineate the underlying mechanism by which PLEC regulates the adhesive properties of ovarian cancer cells, an investigation was conducted into various proteins that are reported to associated with PLEC on *STRING (*https://cn.string-db.org/*)*, The findings revealed a multitude of proteins that interact with PLEC, including RPS15, COL17A1, ITGβ4, SYNE3 and CASP8 etc. Notably, the strongest correlations at the protein level were established between PLEC and COL17A1, ITGβ4, CASP8 at protein level (Fig. 6D). Given that COL17A1 and ITGβ4 play crucial roles in the adhesion process in cancer [23, 24], they were selected for further detailed inquiry in OC.

Subsequently, the target proteins were subjected to immunofluorescence staining and visualized imaging using a confocal laser microscope. The results revealed predominant colocalization of PLEC with COL17A1 and ITGβ4 mostly in both two cell lines (Fig. 6E), corroborating our previous findings. The IHC results obtained from resected tumors in vivo strongly demonstrated a significant decrease in the expression levels of COL17A1 and ITGβ4 upon knockdown of PLEC (Fig. 6F, G).

**Fig. 7:**
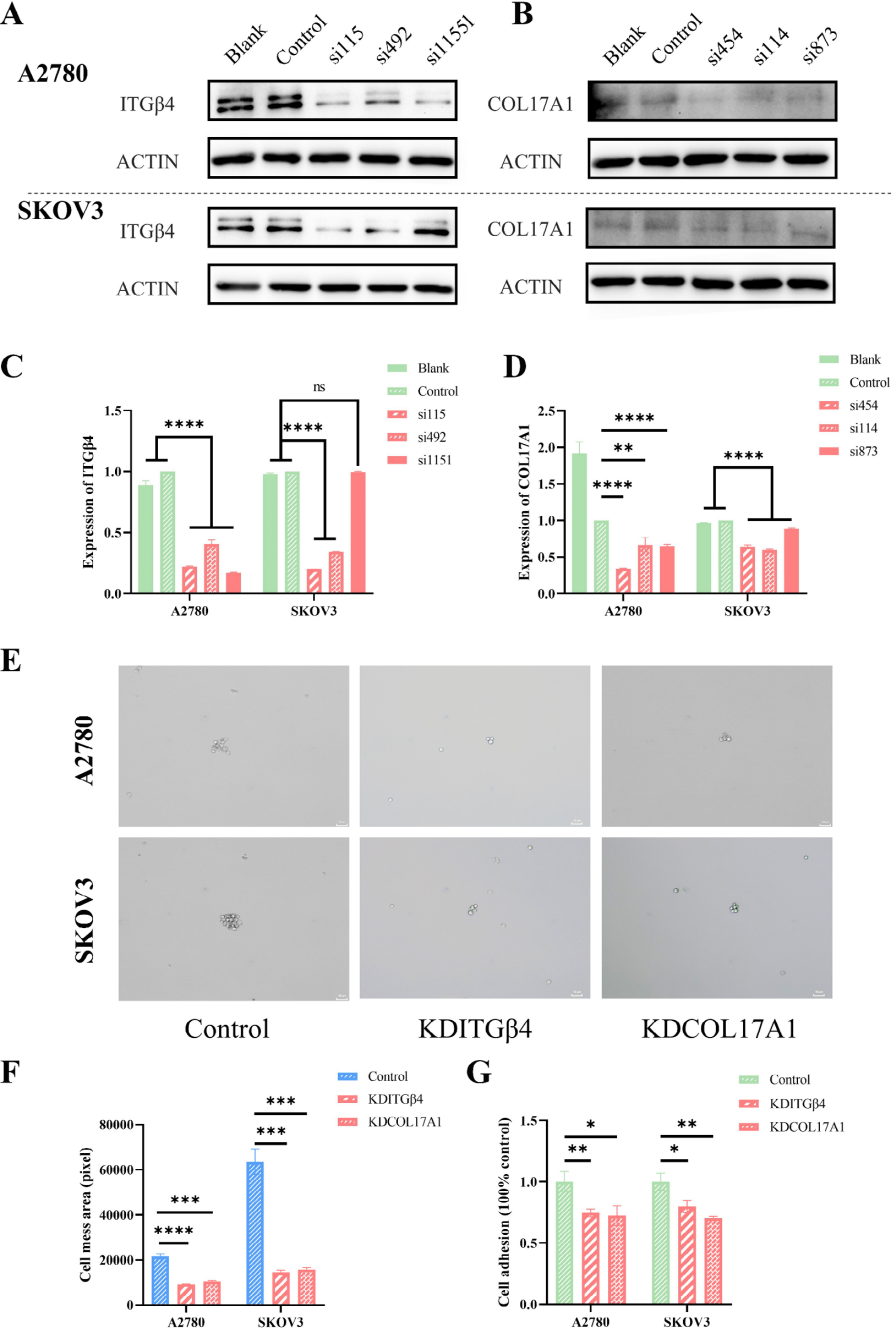
PLEC modulates cell adhesion through its interaction with COL17A1 and ITGβ4. **A**,** B** ITGβ4 and COL17A1 was knock-down in A2780 and SKOV3. Expression of ITGβ4 and COL17A1 was determined by western blotting analysis. **C**,** D** The result of statistical analysis of the grayscale value expressed by ITGβ4 and COL17A1 in A, quantified by imageJ, error bars represent SD. **E** The image fragmented cell monolayers which were detached with dispase and mechanically fragmented by pipetting. **F** The area of those cell fragments in E, quantification by imageJ, error bars represent. **G** Adhesion assay. P-value: *<0.05; **<0.01; ***<0.001; ****<0.0001; ns: no significance, determined by two-tailed Student’s t-test

To verify the specific roles of COL17A1 and ITGβ4 in OC adhesion, we constructed the COL17A1 and ITGβ4 knockdown A2780 and SKOV3 cell lines via siRNA transfection. The results of Western blot confirmed the successful reduction in the expression levels of COL17A1 and ITGβ4 (Fig. 7A-D). Subsequent dissociation assay revealed that the adhesion capacity of A2780 and SKOV3 cells was significantly inhibited following the knockdown of COL17A1 and ITGβ4 (Fig. 7E, F). Besides, cell adhesion assays using a cell adhesion detection kit further affirmed that the adhesion ability of A2780 and SKOV3 cells was significantly reduced after knockdown of COL17A1 and ITGβ4 compared to the Control group (Fig. 7G).

Notably, knockdown of PLEC precipitated a significant decline in the expression levels of COL17A1 and ITGβ4 in both two cell lines, indicating that PLEC may interact with these proteins to modulate their expression. In conclusion, the findings in Figs. 6 and 7supports that PLEC plays a regulatory role in cell adhesion through its interaction COL17A1 and ITGβ4.

## Discussion

Plakins is a family of cytoskeletal binding proteins expressed in a variety of tissues and cells, which anchor the cytoskeletal network to each other [25]. Plectin, a member of plakins family, is a substantial cytoskeletal binding protein with a dumbbell-shaped weighing no less than 500 kDa [9]. It has gained significant attention in recent years due to its pivotal role in various cancer progression [26, 27]. In our previous study, we identified a strong correlation between PLEC and OC, suggesting its potential as a diagnostic biomarker for OC [16, 28]. However, further research in this area remains vacant. Therefore, we conducted additional investigations to elucidate the precise role of PLEC in in the progression of OC.

In this study, our analysis of tissue microarrays revealed a significantly higher expression level of PLEC in OC tumor tissues compared to normal tissues, providing further evidence for the involvement of PLEC in OC progression (Fig. 1). However, the result in Fig. 1D indicated that with the increasing TNM stages, the expression of PLEC did not show a consistent rise according to the H-Score results. This may be caused by two reasons: firstly, the microarrays of ovarian cancer using for IHC was consist of different subtypes of ovarian cancer tissues, including mucinous, serous papillary, endometrioid, clear cell, mucinous papillary and serous carcinomas, the expression score and percentage of PLEC was differential in these subtypes (Table 1), and this was also confirmed in other research results [29]. Secondly, this may be caused by the limitation of tissues number including normal and malignant. Consistent with our result, the expression level of PLEC is significantly altered in various other cancer types, including prostate cancer, colon cancer, and pancreatic cancer [30–32]. Therefore, we generated two PLEC knock-down and Control EOC cell lines for the further investigation (Fig. 2). It has been established that PLEC influences cell proliferation in multiple cell lines due to its impact on mitosis [33]. Our results confirmed a significant decrease in proliferation of PLEC knockdown cell lines both in vitro and in vivo (Fig. 3), providing compelling evidence for the promotion of OC cell proliferation by PLEC.

During tissue cell morphogenesis, post-injury repair and cancer progression, cells usually migrate collectively in groups, chains and sheets [34]. The cytoskeleton which composed of F-actin, MTs and IFs plays a pivotal role in facilitating cell migration, while PLEC exerts its influence on cell migration by interacting with IFs through its C-terminal high-affinity IFs structural domain [35–37]. Previous studies have verified that PLEC promotes cell migration in various diseases and cancers [38, 39], but limited in OC. Therefore, we conducted a series of migration-related experiments, which confirmed the acceleration of OC cell migration and invasion by PLEC both in vitro and in vivo (Figs. 4 and 5), similar to observations made in other cancers [40].

Knockdown of PLEC disrupts adhesion junctions by abolishing wave protein-actin crosslinks [41], indicating of PLEC in cell adhesion at adhesion junctions. Our study further confirmed that that knockdown PLEC in OC cells inhibits cell adhesion (Fig. 6). Osmanagic-Myers S et al. proved that keratinocytes lacking PLEC exhibit enhanced migration compared to normal cells, suggesting a stabilizing function of PLEC in matrix attachment to underlying substrates [42]. Additionally, previous studies have reported the promotion of cancer cell adhesion by PLEC in other types of cancer as well [39, 43, 44], implying its diverse roles across different diseases.

OC metastasis is the main cause of poor clinical outcomes, while colonization is an essential part in OC metastasis, the successful colonization at the target location of the transfer directly determines the success of the metastasis [45]. The enhanced ability of cancer cells to adhere facilitates successful colonization, which is necessary for metastasis to occur, allowing cancer cells to colonize distant sites [46].To elucidate the underlying mechanisms by which PLEC facilitates cell adhesion, our investigation focuses on two adhesion-related molecules: COL17A1 and ITGβ4, based on bioinformatics predictions (Fig. 6D). COL17A1 is a protein involved in the formation of the hemidesmosomes, which anchored to the underlying basement membrane [47]. ITGβ4, a transmembrane adhesion molecule composed of hemidesmosomes, plays a vital role in various cancer cell biology functions [48].Plectin and ITGβ4 have been shown to interact to link with other cytoskeletal networks, thereby contributing to maintain cellular structural organization [27], and previous studies have demonstrated that ITGβ4 and PLEC in promoting prostate cancer progression [30, 49], however, their involvement in OC remains elusive.

In this study, the confocal assay results revealed a significant decrease in the colocalization of PLEC with COL17A1 and ITGβ4, along with a reduction in the expression levels of these two proteins following PLEC knockdown in both two cell lines (Fig. 6E). Subsequently, the IHC assay demonstrated a remarkable decrease in the expression of COL17A1 and ITGβ4 upon PLEC knockdown in vivo (Fig. 6F, G). Upon siRNA-mediated knockdown of COL17A1 and ITGβ4 in A2780 and SKOV3 cells, the detachment assay demonstrated a significant inhibition of cell adhesion (Fig. 7E and F). Additionally, the cell adhesion assays further supported these findings, demonstrating a significant reduction in adhesion capability compared to the control group (Fig. 7G). These findings indicated that PLEC may modulate the expression of COL17A1 and ITGβ4 through its functional interaction with them, and supporting the role of PLEC in cell adhesion.

## Conclusions

In this study, we demonstrated that PLEC exerts a positive influence on the proliferation, migration, invasion and adhesion of ovarian cancer cells. Furthermore, it regulates cell adhesion through its interactions with COL17A1 and ITGβ4. These findings suggest that PLEC has potential as an early diagnostic biomarker and therapeutic target of OC, providing novel insights into the diagnosis and treatment of PLEC in this disease.

## Electronic supplementary material

Below is the link to the electronic supplementary material.

Supplementary Material 1

## Data Availability

Data available on request.

## Abbreviations

OC: Ovarian cancer
PLEC: Plectin
EOC: Epithelial ovarian cancer
FBS: Fetal bovine serum
KD: Knock Down
MOI: Multiplicity of infection
DAPI: 4′,6-diamidino-2-phenylindole
COL17A1: Collagen XVII
ITGβ4: Integrin beta4
DAB: Diaminobenzidine
IHC: Immunohistochemical analysis

## References

[1] Lengyel E. Ovarian cancer development and metastasis[J]. Am J Pathol. 2010;177(3):1053–64. 20651229 10.2353/ajpath.2010.100105 PMC2928939

[2] Siegel RL, Miller KD, Fuchs HE, et al. Cancer statistics, 2022[J]. CA Cancer J Clin. 2022;72(1):7–33. 35020204 10.3322/caac.21708

[3] Li J, Lu J, Xu M et al. ODF2L acts as a synthetic lethal partner with WEE1 inhibition in epithelial ovarian cancer models[J]. J Clin Invest, 2023, 133(2). 10.1172/JCI161544 PMC9843051 36378528

[4] Wang F, Niu Y, Chen K, et al. Extracellular vesicle-packaged circATP2B4 mediates M2 macrophage polarization via miR-532-3p/SREBF1 Axis to promote epithelial ovarian Cancer Metastasis[J]. Cancer Immunol Res. 2023;11(2):199–216. 36512324 10.1158/2326-6066.CIR-22-0410 PMC9896028

[5] Kawakami E, Tabata J, Yanaihara N, et al. Application of Artificial Intelligence for Preoperative Diagnostic and Prognostic Prediction in Epithelial Ovarian Cancer based on blood Biomarkers[J]. Clin Cancer Res. 2019;25(10):3006–15. 30979733 10.1158/1078-0432.CCR-18-3378

[6] Xiao Y, Bi M, Guo H, et al. Multi-omics approaches for biomarker discovery in early ovarian cancer diagnosis[J]. EBioMedicine. 2022;79:104001. 35439677 10.1016/j.ebiom.2022.104001 PMC9035645

[7] Mado K, Chekulayev V, Shevchuk I, et al. On the role of tubulin, plectin, desmin, and vimentin in the regulation of mitochondrial energy fluxes in muscle cells[J]. Am J Physiol Cell Physiol. 2019;316(5):C657–67. 30811221 10.1152/ajpcell.00303.2018

[8] Dicolandrea T, Karashima T, Määttä A, et al. Subcellular distribution of envoplakin and periplakin: insights into their role as precursors of the epidermal cornified envelope[J]. J Cell Biol. 2000;151(3):573–86. 11062259 10.1083/jcb.151.3.573 PMC2185584

[9] Fuchs P, Zörer M, Rezniczek GA, et al. Unusual 5’ transcript complexity of plectin isoforms: novel tissue-specific exons modulate actin binding activity[J]. Hum Mol Genet. 1999;8(13):2461–72. 10556294 10.1093/hmg/8.13.2461

[10] Wiche G. Role of plectin in cytoskeleton organization and dynamics[J]. J Cell Sci. 1998;111(Pt 17):2477–86. 9701547 10.1242/jcs.111.17.2477

[11] Sun D, Leung CL, Liem RK. Characterization of the microtubule binding domain of microtubule actin crosslinking factor (MACF): identification of a novel group of microtubule associated proteins[J]. J Cell Sci. 2001;114(Pt 1):161–72. 11112700 10.1242/jcs.114.1.161

[12] Geerts D, Fontao L, Nievers MG, et al. Binding of integrin alpha6beta4 to plectin prevents plectin association with F-actin but does not interfere with intermediate filament binding[J]. J Cell Biol. 1999;147(2):417–34. 10525545 10.1083/jcb.147.2.417 PMC2174221

[13] Perez SM, Brinton LT, Kelly KA. Plectin in Cancer: from biomarker to therapeutic Target[J]. Cells, 2021, 10(9). 10.3390/cells10092246 PMC8469460 34571895

[14] Wesley T, Berzins S, Kannourakis G, et al. The attributes of plakins in cancer and disease: perspectives on ovarian cancer progression, chemoresistance and recurrence[J]. Cell Commun Signal. 2021;19(1):55. 34001250 10.1186/s12964-021-00726-x PMC8127266

[15] Bausch D, Thomas S, Mino-Kenudson M, et al. Plectin-1 as a novel biomarker for pancreatic cancer[J]. Clin Cancer Res. 2011;17(2):302–9. 21098698 10.1158/1078-0432.CCR-10-0999 PMC3044444

[16] Han Y, Wu J, Yang W, et al. New STAT3-FOXL2 pathway and its function in cancer cells[J]. BMC Mol Cell Biol. 2019;20(1):17. 31221094 10.1186/s12860-019-0206-3 PMC6587274

[17] Mizuta K, Matsubara T, Goto A, et al. Plectin promotes tumor formation by B16 mouse melanoma cells via regulation of Rous sarcoma oncogene activity[J]. BMC Cancer. 2022;22(1):936. 36038818 10.1186/s12885-022-10033-4 PMC9426213

[18] Zhang Q, Zhou X, Wan M, et al. FoxP3-miR-150-5p/3p suppresses ovarian tumorigenesis via an IGF1R/IRS1 pathway feedback loop[J]. Cell Death Dis. 2021;12(3):275. 33723215 10.1038/s41419-021-03554-6 PMC7961150

[19] Kim MJ, Jung D, Park JY et al. GLIS1 in Cancer-Associated fibroblasts regulates the Migration and Invasion of Ovarian Cancer Cells[J]. Int J Mol Sci, 2022, 23(4). 10.3390/ijms23042218 PMC8874490 35216340

[20] Jiang GJ, You XG, Fan TJ. Carteolol triggers senescence via activation of β-arrestin-ERK-NOX4-ROS pathway in human corneal endothelial cells in vitro[J]. Chem Biol Interact. 2023;380:110511. 37120125 10.1016/j.cbi.2023.110511

[21] Zrelski MM, Hösele S, Kustermann M et al. Plectin Deficiency in fibroblasts deranges intermediate filament and Organelle Morphology, Migration, and Adhesion[J]. J Invest Dermatol, 2023. 10.1016/j.jid.2023.08.020 37716646

[22] Valencia RG, Walko G, Janda L, et al. Intermediate filament-associated cytolinker plectin 1c destabilizes microtubules in keratinocytes[J]. Mol Biol Cell. 2013;24(6):768–84. 23363598 10.1091/mbc.E12-06-0488 PMC3596248

[23] Ohta Y, Fujii M, Takahashi S, et al. Cell-matrix interface regulates dormancy in human colon cancer stem cells[J]. Nature. 2022;608(7924):784–94. 35798028 10.1038/s41586-022-05043-y

[24] Mohanty A, Nam A, Srivastava S, et al. Acquired resistance to KRAS G12C small-molecule inhibitors via genetic/nongenetic mechanisms in lung cancer[J]. Sci Adv. 2023;9(41):eade3816. 37831779 10.1126/sciadv.ade3816 PMC10575592

[25] Hu L, Huang Z, Wu Z et al. Mammalian plakins, Giant Cytolinkers: versatile biological functions and roles in Cancer[J]. Int J Mol Sci, 2018, 19(4). 10.3390/ijms19040974 PMC5979291 29587367

[26] Amiri A, Dietz C, Rapp A, et al. The cyto-linker and scaffolding protein plectin mis-localization leads to softening of cancer cells[J]. Nanoscale. 2023;15(36):15008–26. 37668423 10.1039/d3nr02226a

[27] Gao K, Gao Z, Xia M, et al. Role of plectin and its interacting molecules in cancer[J]. Med Oncol. 2023;40(10):280. 37632650 10.1007/s12032-023-02132-4

[28] Prechova M, Korelova K, Gregor M. Plectin[J]. Curr Biol. 2023;33(4):R128–30. 36854266 10.1016/j.cub.2022.12.061

[29] Dasa SS, K, Diakova G, Suzuki R, et al. Plectin-targeted liposomes enhance the therapeutic efficacy of a PARP inhibitor in the treatment of ovarian cancer[J]. Theranostics. 2018;8(10):2782–98. 29774075 10.7150/thno.23050 PMC5957009

[30] Wenta T, Schmidt A, Zhang Q, et al. Disassembly of α6β4-mediated hemidesmosomal adhesions promotes tumorigenesis in PTEN-negative prostate cancer by targeting plectin to focal adhesions[J]. Oncogene. 2022;41(30):3804–20. 35773413 10.1038/s41388-022-02389-5 PMC9307480

[31] Zhu X, Jiang X, Zhang Q, et al. TCN1 Deficiency inhibits the malignancy of Colorectal Cancer cells by regulating the ITGB4 Pathway[J]. Gut Liver. 2023;17(3):412–29. 35686504 10.5009/gnl210494 PMC10191790

[32] Wang Q, Yan H, Jin Y, et al. A novel plectin/integrin-targeted bispecific molecular probe for magnetic resonance/near-infrared imaging of pancreatic cancer[J]. Biomaterials. 2018;183:173–84. 30172243 10.1016/j.biomaterials.2018.08.048

[33] Steinböck FA, Wiche G. Plectin: a cytolinker by design[J]. Biol Chem. 1999;380(2):151–8. 10195422 10.1515/BC.1999.023

[34] Mayor R, Etienne-Manneville S. The front and rear of collective cell migration[J]. Nat Rev Mol Cell Biol. 2016;17(2):97–109. 26726037 10.1038/nrm.2015.14

[35] Leube RE, Moch M, Windoffer R. Intermediate filaments and the regulation of focal adhesion[J]. Curr Opin Cell Biol. 2015;32:13–20. 25460777 10.1016/j.ceb.2014.09.011

[36] Wiche G, Osmanagic-Myers S, Castañón MJ. Networking and anchoring through plectin: a key to IF functionality and mechanotransduction[J]. Curr Opin Cell Biol. 2015;32:21–9. 25460778 10.1016/j.ceb.2014.10.002

[37] Seltmann K, Cheng F, Wiche G, et al. Keratins stabilize hemidesmosomes through regulation of β4-Integrin Turnover[J]. J Invest Dermatol. 2015;135(6):1609–20. 25668239 10.1038/jid.2015.46

[38] Buckup M, Rice MA, Hsu EC, et al. Plectin is a regulator of prostate cancer growth and metastasis[J]. Oncogene. 2021;40(3):663–76. 33219316 10.1038/s41388-020-01557-9 PMC8078627

[39] Xu R, He S, Ma D et al. Plectin downregulation inhibits Migration and suppresses Epithelial Mesenchymal Transformation of Hepatocellular Carcinoma Cells via ERK1/2 Signaling[J]. Int J Mol Sci, 2022, 24(1). 10.3390/ijms24010073 PMC9820339 36613521

[40] Sutoh Yoneyama M, Hatakeyama S, Habuchi T, et al. Vimentin intermediate filament and plectin provide a scaffold for invadopodia, facilitating cancer cell invasion and extravasation for metastasis[J]. Eur J Cell Biol. 2014;93(4):157–69. 24810881 10.1016/j.ejcb.2014.03.002

[41] Osmanagic-Myers S, Rus S, Wolfram M, et al. Plectin reinforces vascular integrity by mediating crosstalk between the vimentin and the actin networks[J]. J Cell Sci. 2015;128(22):4138–50. 26519478 10.1242/jcs.172056 PMC4712781

[42] Osmanagic-Myers S, Gregor M, Walko G, et al. Plectin-controlled keratin cytoarchitecture affects MAP kinases involved in cellular stress response and migration[J]. J Cell Biol. 2006;174(4):557–68. 16908671 10.1083/jcb.200605172 PMC2064261

[43] Katada K, Tomonaga T, Satoh M, et al. Plectin promotes migration and invasion of cancer cells and is a novel prognostic marker for head and neck squamous cell carcinoma[J]. J Proteom. 2012;75(6):1803–15. 10.1016/j.jprot.2011.12.018 22245045

[44] Mcinroy L, Määttä A. Plectin regulates invasiveness of SW480 colon carcinoma cells and is targeted to podosome-like adhesions in an isoform-specific manner[J]. Exp Cell Res. 2011;317(17):2468–78. 21821021 10.1016/j.yexcr.2011.07.013

[45] Śliwa A, Szczerba A, Pięta PP et al. A recipe for successful metastasis: transition and migratory modes of Ovarian Cancer Cells[J]. Cancers (Basel), 2024, 16(4). 10.3390/cancers16040783 PMC10886816 38398174

[46] Paolillo M, Schinelli S. Extracellular matrix alterations in metastatic Processes[J]. Int J Mol Sci, 2019, 20(19). 10.3390/ijms20194947 PMC6802000 31591367

[47] Kozawa K, Sekai M, Ohba K, et al. The CD44/COL17A1 pathway promotes the formation of multilayered, transformed epithelia[J]. Curr Biol. 2021;31(14):3086–e30977. 34087104 10.1016/j.cub.2021.04.078

[48] Yang H, Xu Z, Peng Y et al. Integrin β4 as a potential diagnostic and therapeutic tumor Marker[J]. Biomolecules, 2021, 11(8). 10.3390/biom11081197 PMC8394641 34439865

[49] Koivusalo S, Schmidt A, Manninen A et al. Regulation of kinase signaling pathways by α6β4-Integrins and plectin in prostate Cancer[J]. Cancers (Basel), 2022, 15(1). 10.3390/cancers15010149 PMC9818203 36612146

