# Supplementary material for: Plectin, a novel regulator in migration, invasion and adhesion of ovarian cancer
Source: Cell Biosci. 2025 Feb 6;15:15. doi: 10.1186/s13578-025-01349-2 (PMC11804098; doi:10.1186/s13578-025-01349-2)
Supplement: Supplementary file 1 — Supplementary Material 1 [file 13578_2025_1349_MOESM1_ESM.docx]

**Fig 2.A-A2780 ACTIN**


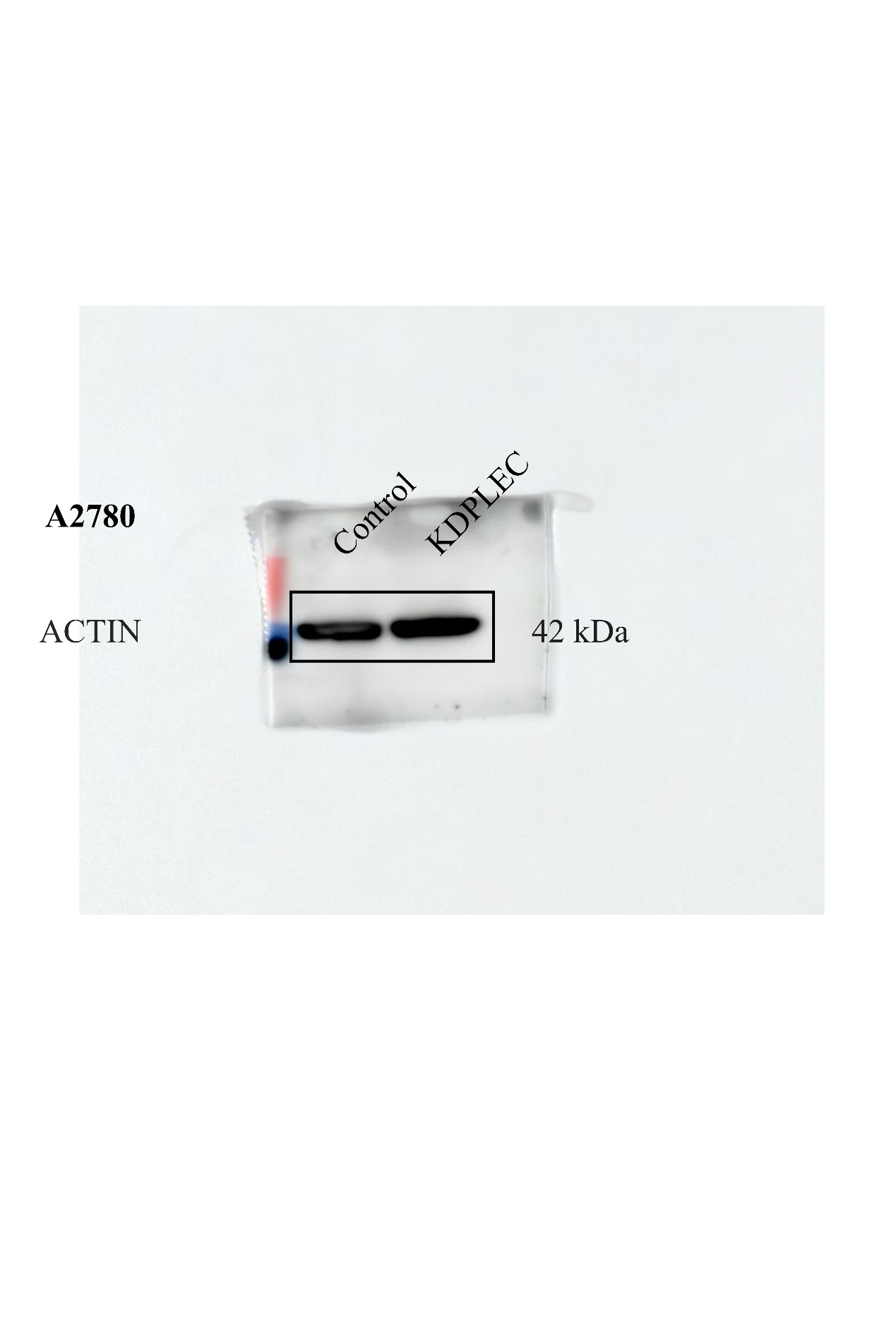


**Fig 2.A-A2780 plectin**


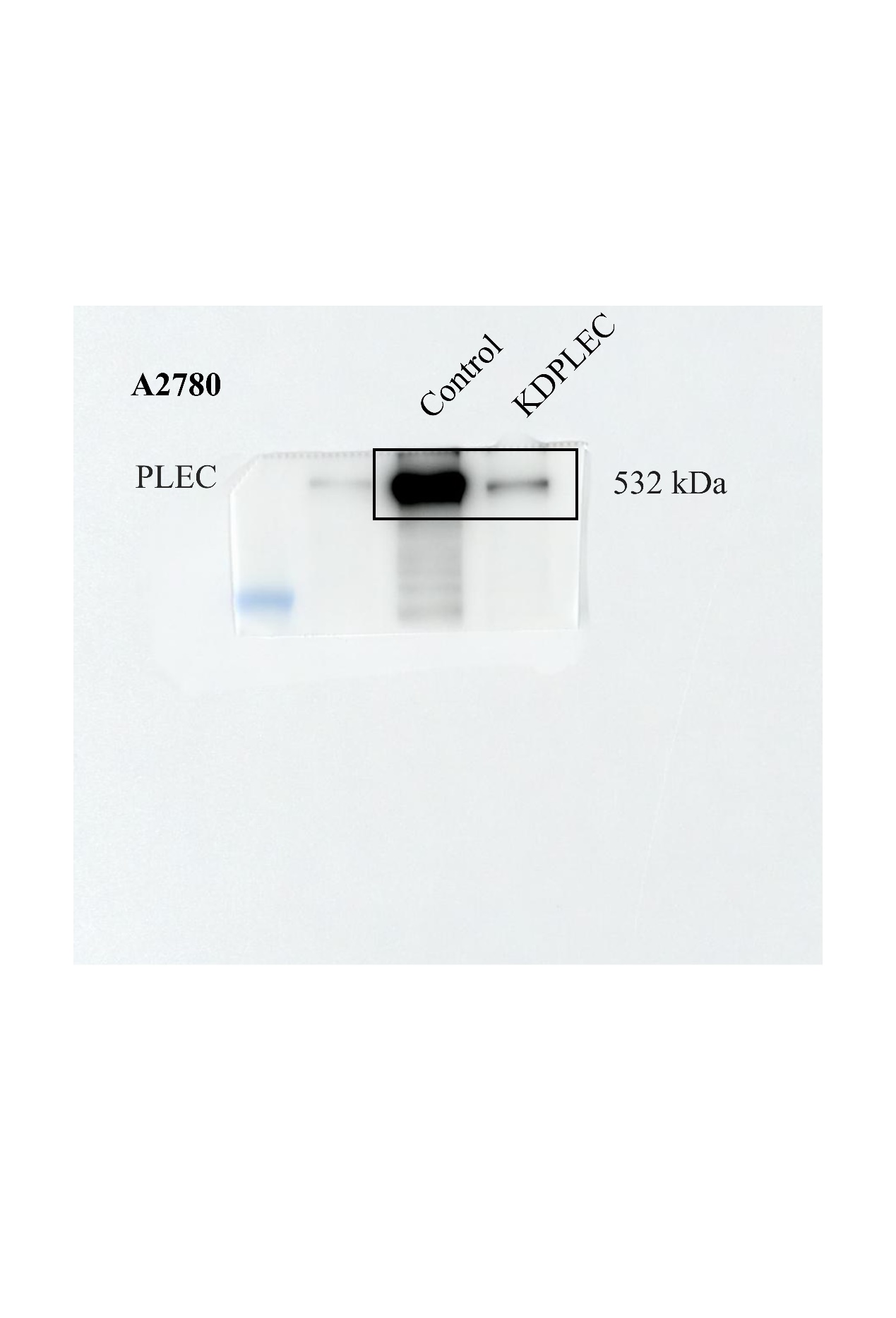


**Fig 2.A-SKOV3 actin**


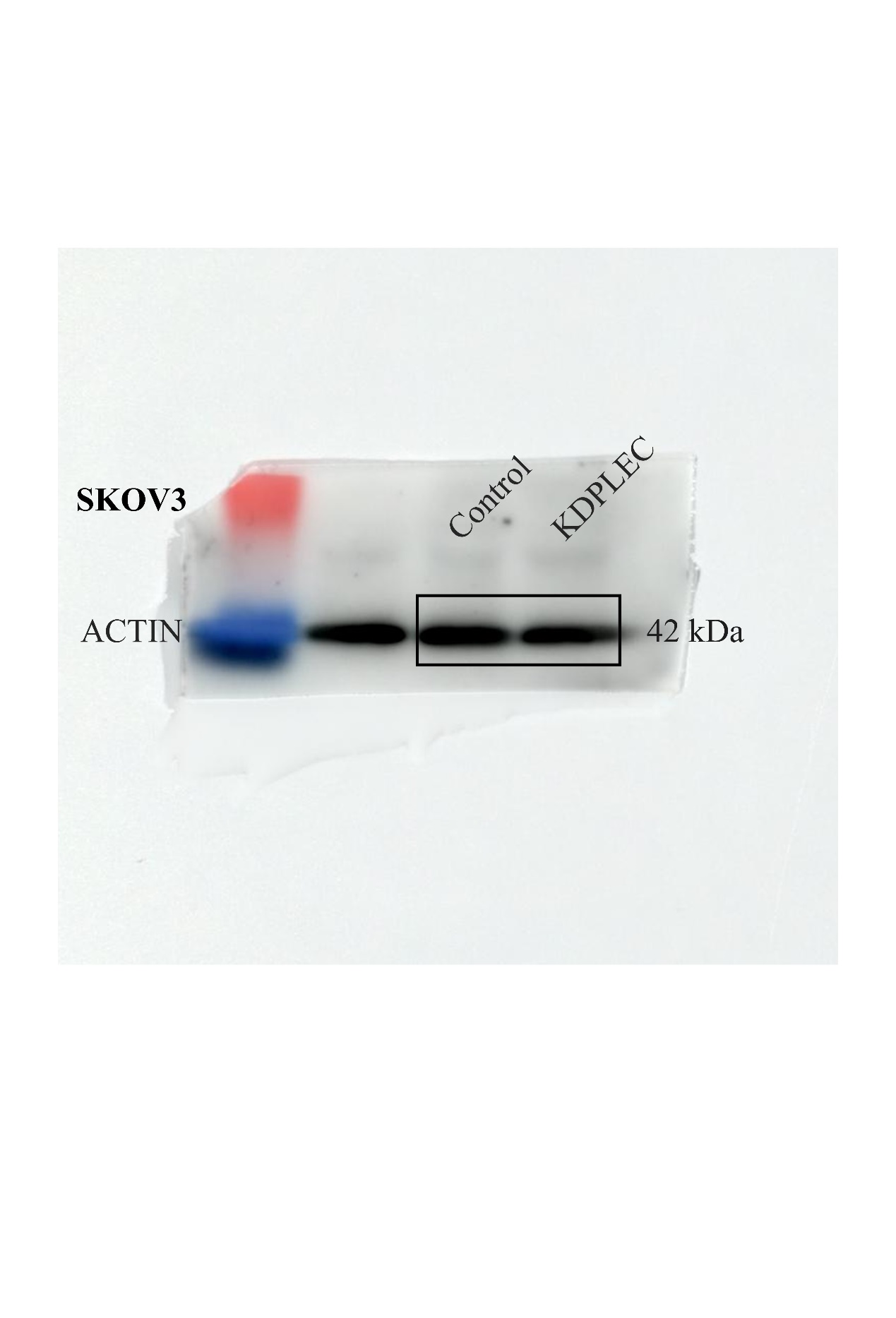


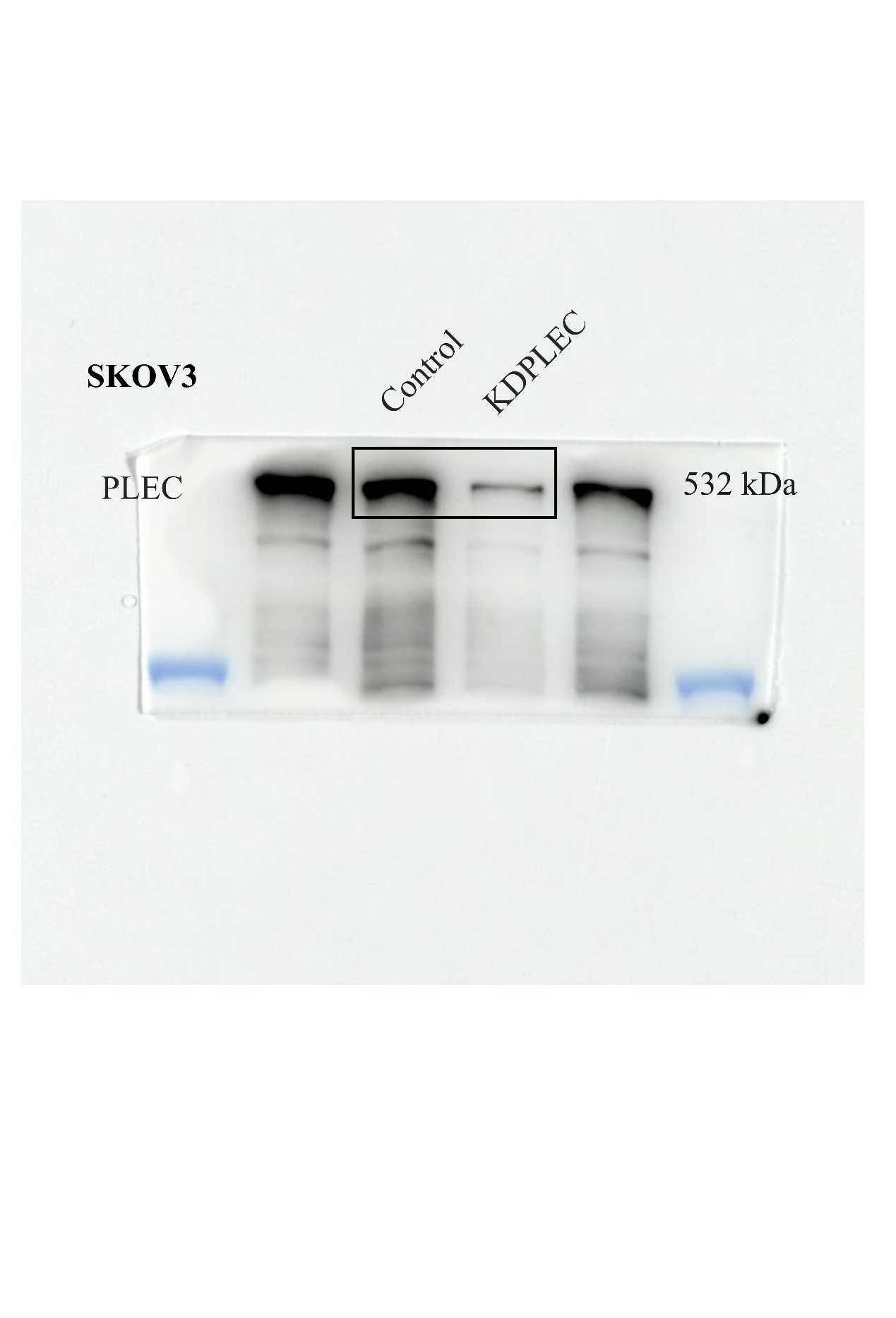
**Fig 2.A-SKOV3 plectin**

**
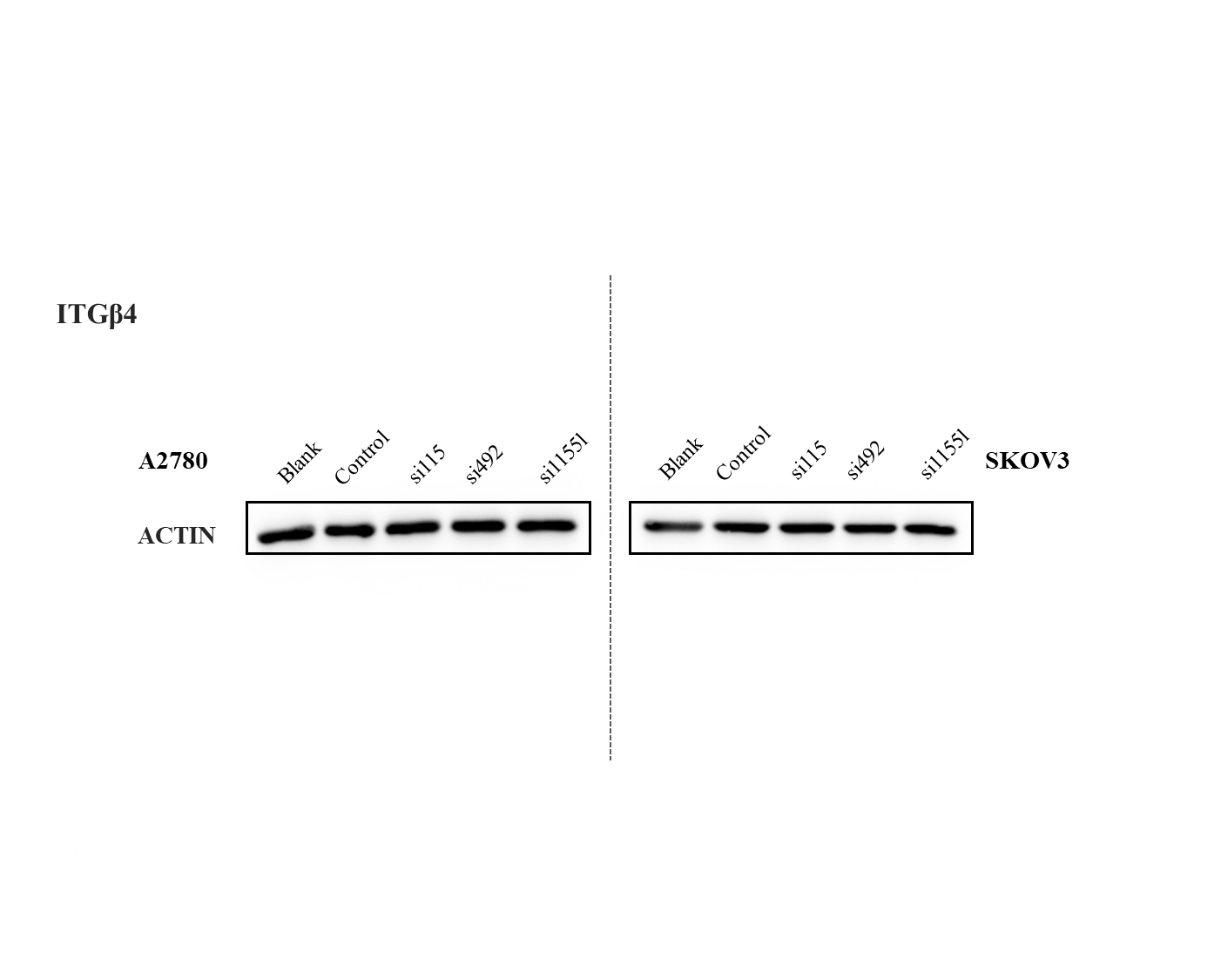
Fig 7.A-ACTIN**

**
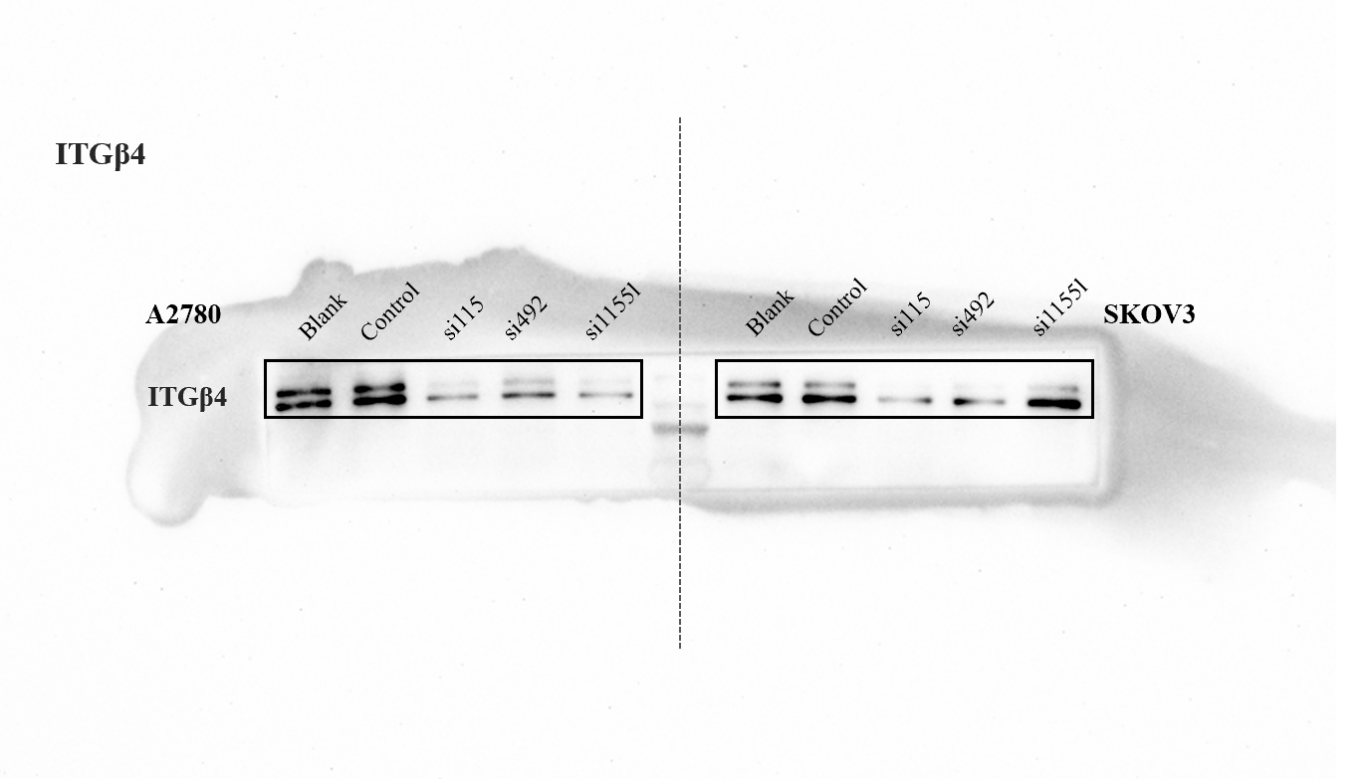
Fig 7.A-ITGβ4**

**
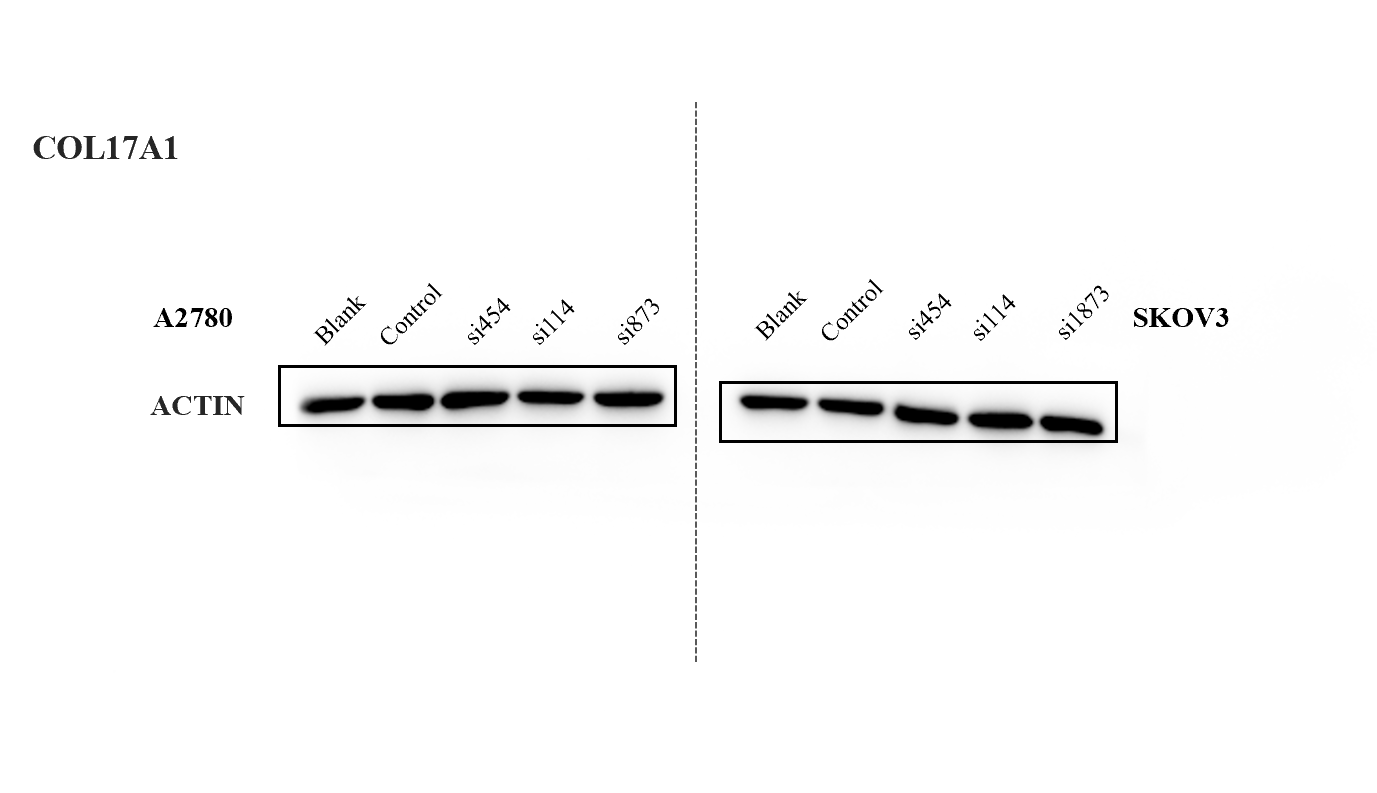
Fig 7.B-ACTIN**

**
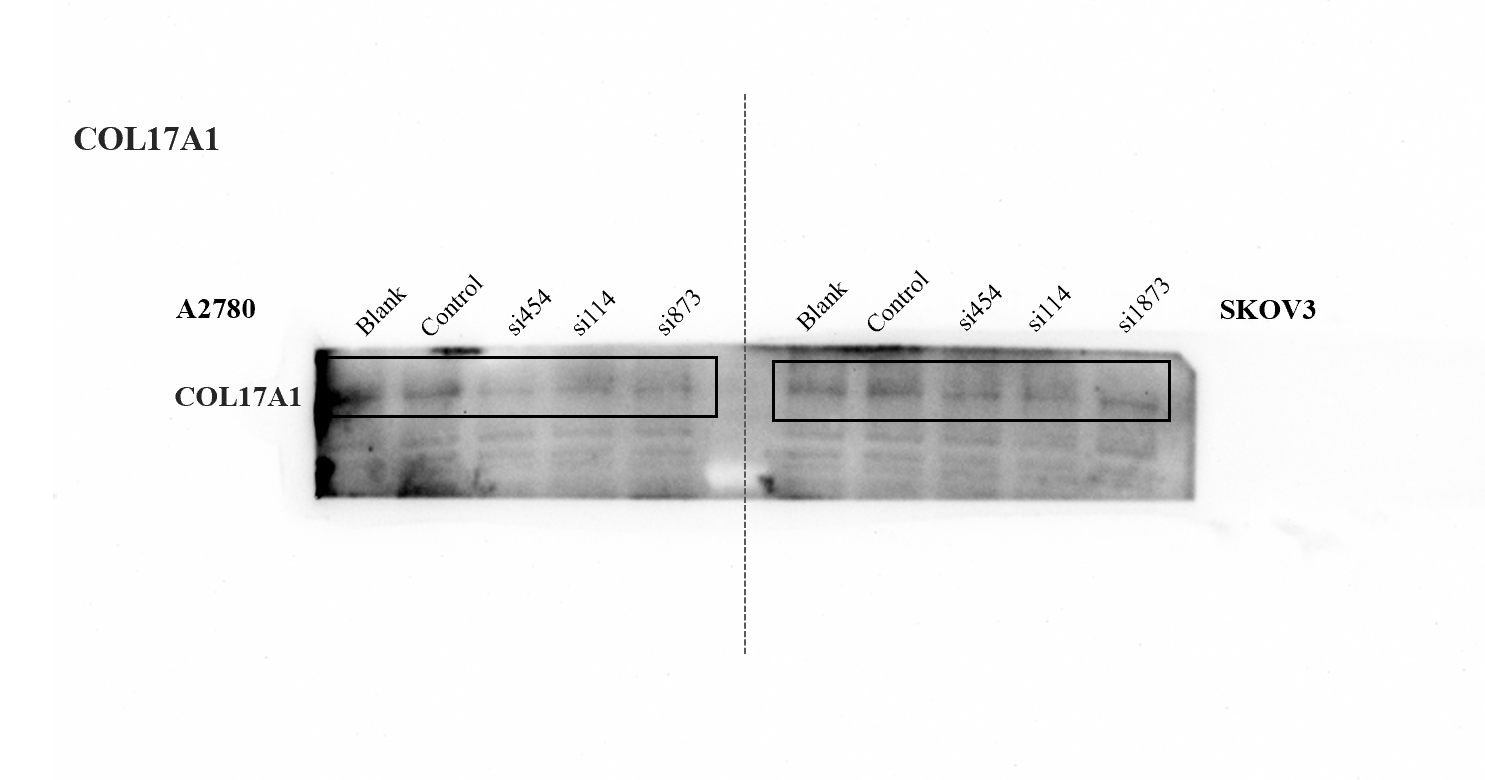
Fig 7.B-COL17A1**
